# Moving to Beat Anxiety: Epidemiology and Therapeutic Issues with Physical Activity for Anxiety

**DOI:** 10.1007/s11920-018-0923-x

**Published:** 2018-07-24

**Authors:** Aaron Kandola, Davy Vancampfort, Matthew Herring, Amanda Rebar, Mats Hallgren, Joseph Firth, Brendon Stubbs

**Affiliations:** 1 Physiotherapy Department, South London and Maudsley NHS Foundation Trust, Denmark Hill, London, SE5 9RS UK; 2 KU Leuven Department of Rehabilitation Sciences, Leuven, Belgium; 3 KU Leuven Department of Neurosciences, UPC KU Leuven, Kortenberg, Belgium; 4 Department of Physical Education and Sport Sciences, University of Limerick, Limerick, Ireland; 5 Health Research Institute, University of Limerick, Limerick, Ireland; 6 Physical Activity Research Group, School of Health, Medical and Applied Sciences, Central Queensland University, Rockhampton, QLD Australia; 7 Department of Public Health Sciences, Karolinska Institute, Stockholm, Sweden; 8 NICM Health Research Institute, School of Science and Health, University of Western Sydney, Sydney, Australia; 9 Division of Psychology and Mental Health, University of Manchester, Manchester, UK; 10 Department of Psychological Medicine, Institute of Psychiatry, Psychology and Neuroscience, King’s College London, London, UK

**Keywords:** Exercise, Fitness, Depression, Cardiovascular disease, Stress, Mental health

## Abstract

**Purpose of Review:**

The purpose of this paper was to provide a comprehensive narrative review of the relationship between physical activity (PA) and anxiety and the rationale for including it as a treatment option for anxiety disorders. Several gaps in the literature are highlighted alongside recommendations for future research.

**Recent Findings:**

PA in the general population has established efficacy in preventing and managing cardiovascular disease and improving wellbeing. Recent epidemiological data further suggests that people who are more active may be less likely to have anxiety disorders. In addition, evidence from systematic reviews of randomised control trials suggests that exercise training, a subset of PA, can reduce symptoms in anxiety and stress-related disorders, such as post-traumatic stress disorder, agoraphobia and panic disorder.

**Summary:**

Anxiety disorders are common, burdensome and costly to individuals and wider society. In addition to the profound negative impact on individuals’ wellbeing and functioning, they are associated with worsened physical health, including a higher risk for cardiovascular diseases and premature mortality. Although pharmacotherapy and psychological interventions are helpful for many, these treatment approaches are not effective for everyone and are insufficient to address common physical health complications, such as the elevated risk of cardiovascular disease. Given the combined anxiolytic and physical health benefits of increased activity, PA presents a promising additional treatment option for people with anxiety disorders. However, there remain key gaps in the literature regarding the mechanisms underlying the effects of PA, optimal PA protocols, methods of improving adherence and the importance of physical fitness. These must be addressed for PA to be successfully implemented in mental health services.

## Introduction

Anxiety disorders are a heterogeneous cluster of common mental health disorders typically characterised by hyperarousal, excessive fear and worry [1]. Although anxiety disorders are generally characterised by feelings of anxiety, symptoms can manifest themselves differently depending on the disorder subtype. Subtypes of anxiety disorders include generalised anxiety disorder (GAD), social phobia, panic disorder, specific phobias, agoraphobia, separation anxiety disorder and selective mutism. Anxiety disorders are highly prevalent with global estimates ranging from 3.8 to 25% across countries, with estimates of prevalence rates as high as 70% in people with chronic health conditions [2]. These widespread disorders have a debilitating impact on people’s daily functioning, quality of life and wellbeing [3]⁠. Data from the Global Burden of Disease study suggests that anxiety disorders are the sixth leading cause of global disability [4]⁠, accounting for 26.8 million disability-adjusted life years [5]⁠. Anxiety disorders are highly comorbid with other mental disorders [6]⁠, including depression [7]⁠ and substance use disorders [8]⁠. Worryingly, anxiety disorders are also associated with elevated cardiovascular risk factors such as high blood pressure [9]⁠, a higher rate of cardiovascular disease [10•]⁠ and with premature mortality [11–14]⁠.

## Current Treatment Approaches

Anxiety disorders are typically treated using pharmacological agents such as selective serotonin reuptake inhibitors (SSRIs), serotonin-norepinephrine reuptake inhibitors (SNRIs) or benzodiazepines [15, 16]⁠, cognitive behavioural therapy (CBT) [17]⁠ or a combination of both [18]⁠. These traditional approaches can be effective in reducing anxiety disorder symptoms, but up to one-third of patients do not respond to treatment [19, 20] which can lead to treatment dropout, poorer outcomes and functioning [21]⁠. To address this issue, multimodal approaches that combine pharmacotherapy with forms of CBT have been developed, but have yet to produce any discernible improvements in efficacy [18, 22]⁠. A number of practical (e.g. availability of therapists) and financial barriers also exist to the effective delivery of these types of therapy [23]⁠, particularly in low- and middle-income countries [24•, 25•]⁠. This greatly limits the universal availability for these types of therapy across all population groups.

## Treatment Gap: the Need for Interventions to Improve Physical Health

Beyond treating the symptoms of anxiety disorders, it is also unclear to what extent traditional approaches are able to manage wider issues of physical comorbidity, which is common in people with anxiety disorders [26]⁠. For example, people with anxiety disorder are 52% more likely to develop a cardiovascular disease than the general population [10(].⁠ Given that the physical health problems associated with anxiety disorders and comorbidities, and not directly caused by anxiety, improving the poor physical health, outcomes associated with anxiety disorders cannot be achieved using pharmacotherapy or CBT alone. While it is essential that interventions are offered to reduce the mental health symptoms, it is also essential that interventions are developed to prevent the onset and manage physical health problems in people with anxiety disorders [27]⁠.

## Potential for Physical Activity

Physical activity (PA)-based interventions represent a novel approach that have demonstrated efficacy in treating symptoms of several mental health conditions ranging from psychosis (e.g. [28, 29])⁠ to dementia (e.g. [30])⁠. It is worth noting that the broader concept of PA can be distinguished from physical exercise, which refers to a form of PA that is structured to improve physical fitness [31]⁠. In this paper, PA will be used to refer to both incidental activity (e.g. walking to the shops) and physical exercise (e.g. walking on a treadmill at the gym), given that there is a lack of discernible empirical evidence that the anxiolytic benefits of activity are dependent on fitness changes [32••]⁠. However, the vast majority of empirical research in the field have used exercise-based interventions specifically.

Most notably, exercise-based interventions have been consistently demonstrated as having an antidepressant effect in people with depression [33, 34]⁠, with some studies reporting a treatment efficacy comparable to antidepressant medications or psychotherapy [35–37]⁠. In such mental health conditions, PA has been shown to have wider benefits to peoples’ wellbeing, including an improved quality of life, less psychological distress and, importantly, improved physical health [28, 29, 38–43]⁠. In addition to having few adverse side effects, PA may have further benefits for daily functioning as research has found exercise training to produce improved neurocognitive performances in people with mental health conditions such as schizophrenia or depression [44, 45].

Recent work suggests that PA may also be beneficial for the treatment of anxiety disorders [46••]⁠. Given the potential for PA to improve physical as well as mental health, PA-based interventions may be an important trans-diagnostic tool with an array of broader benefits to people with anxiety disorders. The following sections will provide a brief overview of the evidence supporting PA as a treatment for anxiety disorders and outline several gaps in the literature to be addressed.

## Physical Activity and Anxiety: Current Evidence

Several studies of the general population have found that people who engage in more PA have a reduced risk of being diagnosed with an anxiety disorder and less frequent and severe anxiety symptoms [47–52]⁠. Conversely, physical inactivity has been identified as a risk factor for the development of anxiety [53]⁠ as well as for other conditions such as depression [54–56]⁠. This evidence suggests that engagement in PA appears to be protective for anxiety symptoms and disorders in the general population.

Despite the strong basis of evidence for its effectiveness in treating depression [33]⁠ and the commonality of anxiety disorders co-occurring with depression [57, 58], there has been comparatively little work on the use of PA to treat anxiety disorders. Nonetheless, recent reviews have found PAs, typically exercise-based interventions, are effective as standalone or adjunctive therapies for reducing anxiety symptoms, with effect sizes ranging from small to moderate in people with [32••, 46••, 59–61]⁠ and without [32••, 62–65]⁠ a diagnosed anxiety disorder. Exercise-based interventions have been shown to reduce symptoms in trauma and stress-related disorders such as in patients with post-traumatic stress disorder (PTSD) [66]⁠. Even acute bouts of exercise have been shown to have a small, positive effect on reducing symptoms of state anxiety [67]⁠. An innovative recent study found that aerobic exercise in addition to cognitive behavioural therapy improved symptoms in people with panic disorder and agoraphobia, possibly acting through the “breathing hypotheses” [68]. PA-based interventions have also been found to reduce anxiety symptoms in patients with chronic physical health conditions [69]⁠. Of note, the majority of these studies have used aerobic exercise, and as a result, little is known about the mental health benefits of resistance training [70]⁠. However, a recent review suggests that resistance training has a small-moderate impact on reducing anxiety symptoms in clinical and non-clinical populations [71•].

The available evidence strongly suggests that PA interventions have a broadly reductive effect on anxiety symptoms across clinical and non-clinical population groups. The low-cost, low-risk nature of PA means that such interventions could be a useful treatment option for anxiety. Its antidepressant properties also allow for PA to be used as a trans-diagnostic treatment for people with a co-morbid affective disorder.

Given the elevated physical health risks associated with anxiety disorders [9]⁠, its capacity to reduce cardiovascular risks is amongst the most important aspects of PA in the treatment of anxiety disorders. However, studies of PA-based interventions in anxiety disorders rarely record physical health outcomes [32••],⁠ and little work has been conducted to directly assess the capacity for PA to reduce physical health risks in people with anxiety disorders. Future research should seek to address these shortcomings by developing RCTs which measure both the physical and mental health outcomes associated with PA-based interventions in people with anxiety disorders.

This is a relatively novel area of research compared to more established approaches such as psychotherapy. There are also fewer financial resources available for this type of research relative to that of mainstream pharmacotherapy. As a result, more high-quality randomised controlled trials (RCTs) are still required to address certain gaps in the literature to facilitate the most effective and appropriate use of PA in the treatment of anxiety. The following sections will highlight some key gaps in the literature that should be addressed by future research.

## Mechanisms of Action

PA may act through a range of different physiological and psychological mechanisms, but the precise mechanism by which PA reduces anxiety is less clear. Potential mechanisms could include the role of PA plays in the regulation of stress responses through the hypothalamic-pituitary-adrenal (HPA) axis or glucocorticoids circulation [72, 73]⁠. PA also stimulates a broad range of neurogenic processes important for proper brain functioning, such as up-regulating growth factors like brain-derived neurotrophic factor (BDNF), and stimulating neurogenesis and angiogenesis [74, 75]⁠. By promoting the functioning of brain regions that have particular relevance to anxiety or stress-related conditions, PA could help to reduce symptoms of anxiety. For example, the hippocampus is a region intrinsic to regulating feedback of stress responses from the HPA [76, 77]⁠ and may be impaired in people with PTSD [78, 79]⁠. PA has a particularly potent impact on improving hippocampal functioning [75]⁠, which may support stress response regulation and reduce PTSD symptoms. Moreover, a recent meta-analysis of human RCTs demonstrated that exercise may improve left hippocampal volume in particular [80].

Another possible mechanism through which PA may improve symptoms is the potential to influence the inflammatory system, which has been implicated in the aetiology and severity of symptoms in anxiety disorders. Specifically, anxiety disorders are associated with elevated levels of the pro-inflammatory cytokine C-reactive protein (CRP), which can cause chronic inflammation and contribute towards the disorder development [81]⁠. Prolonged HPA dysfunction can also impair anti-inflammatory responses to glucocorticoids, causing further inflammation [82]⁠. PA is known to have anti-inflammatory properties and may exert a positive impact on treating anxiety disorders by mediating inflammatory pathways [83]⁠. Other recent evidence suggests that PA may also operate through the endocannabinoid system. For example, PA has been shown to up-regulate circulating endocannabinoids such as anandamide, which can produce anxiolytic effects by regulating other neurotransmitters, such as dopamine [84]⁠. Other systems such as the monoamine or endogenous opioid system have also been suggested but a comprehensive overview of all the potential physiological mechanisms of PA is beyond the scope of the current paper and has been covered in other reviews (e.g. [72, 73])⁠.

PA may also reduce symptoms of anxiety through psychological mechanisms, such as anxiety sensitivity. Several studies have found PA to reduce anxiety sensitivity by reliably producing similar physiological responses as experienced with high anxious states, such as increased heart rate, but without the negative, anxious feelings that people with anxiety disorders have grown to associate with such physiological arousal [85, 86]⁠. By encouraging people to associate these physiological experiences with pleasant enjoyable or non-threatening experiences, it may help to reduce the anticipated dread of these somatic symptoms and increase the tolerance and management of them [85, 86]⁠.

The concept of habituating people to anxiety symptoms is also a potential mechanism through which CBT may take its effect [87]⁠. Similarly, PA may promote self-efficacy for coping with anxiety through producing mastery experiences [88]⁠. Poor self-beliefs such as efficacy and self-esteem can be experienced as part of anxiety disorders, so feelings of achievement and mastery through PA can help to enhance positive self-beliefs and buffer from negative self-perception worries [89]⁠.

It is currently unclear as to which physiological and psychological mechanisms PA produces an anxiolytic effect. Future research should focus on investigating the factors through which PA reduces anxiety symptoms as this may have direct implications for how interventions are designed. For example, should research demonstrate that neurogenic processes play a key role in PA producing anxiolytic effects, the minimum length of an intervention may be influenced by the time in which these processes take to impact brain function. A clearer understanding of these mechanisms may also provide the opportunity to elicit a stronger treatment effect by combining PA with other types of therapy which operate through overlapping, or complementary pathways [90]⁠.

Furthering our understanding as to exactly how PA interacts with anxiety symptoms may help to inform PA-based interventions that are customised to a patient’s biological, psychological or symptom profile. As well as improving the design of PA interventions, this research would also contribute towards our understanding of the aetiology of anxiety. Such findings could be used to develop more targeted pharmacotherapies or public health strategies to prevent anxiety disorders.

## Optimal Physical Activity Protocols

There is currently a lack of clarity over the frequency, intensity, duration and type of PA parameters that most effectively reduce anxiety symptoms. For example, there is a distinct lack of RCTs comparing the additive or interactive impacts of aerobic and resistance activity on anxiety symptoms within the same study [70]⁠. This has led to a lack of clarity over whether aerobic or resistance exercises have divergent effects on anxiety symptoms. There is some recent RCT evidence to suggest that the impact of aerobic and resistance exercises may differ as to which symptoms and underlying constructs of anxiety disorders they affect [91, 92•]. For example, LeBouthillier and colleagues [92•]⁠ demonstrated that while both exercise modalities were efficacious in improving disorder status, only aerobic exercise improved general psychological distress and anxiety, while only resistance training improved anxiety sensitivity, distress tolerance and intolerance of uncertainty.

However, another study comparing acute bouts of aerobic and resistance exercise found them to have a significant, but equivocal impact on reducing anxiety sensitivity and no other anxiety constructs [91]⁠. An earlier study reported that resistance exercise elicited notably greater reductions in signs and symptoms of GAD, than aerobic exercise [93]⁠. In addition to these mixed findings, drawing any firm conclusions from the available literature is further complicated by the scarcity of studies that sufficiently match aerobic and resistance exercise protocols on active features of the exercise stimulus (e.g. time actively engaged in exercise, work completed during exercise sessions, and progression in load) to facilitate a more rigorous exercise [71•]⁠.

Along with the comparative efficacy of aerobic and resistance activity, other aspects of PA have been equally understudied in terms of their relative efficacy such as sport vs. leisure-time activity or individual vs. group-based exercise. To provide clarity, RCTs using robust and consistent methodologies could be used to establish the relative effectiveness and differences between types of PA.

Further to this, establishing a dose-response relationship between the frequency or intensity of PA with anxiolytic effects represents another important goal for future research. Determining the most beneficial types of activity for people with anxiety disorders is challenging, as these and likely idiosyncratic processes, unique to each person [94]⁠ and specific details on the PA protocols used in studies, are not consistently reported [32••, 71•, 95]⁠. Moreover, there is a lack of variability in the intensity or frequency of PA used across studies of anxieties making it difficult to establish differences in efficacy between them [46••]⁠. A small study (*N* = 18) tested a dose-response relationship by comparing the anxiolytic impact of varying intensity exercise and demonstrated that there were less panic reactions to CO_2_ in patients that performed moderate-to-vigorous intensity in contrast to those who performed very light intensity activity [96]⁠. However, the study is likely to be underpowered and is not designed for causal inferences. More research is needed to test the replicability and generalizability of these findings.

Robust RCTs that manipulate and compare different intensities, frequencies or durations of PA on anxiety symptoms are required to elucidate a dose-response relationship [32••]⁠. Understanding this relationship will facilitate the development of PA-based interventions that are tailored to the treatment of anxiety symptoms, instead of having to rely on public health guidelines designed for physical health such as the American College of Sports Medicine [97]⁠.

## Adherence to Interventions

In addition to maximising efficacy research, establishing PA protocols tailored to mental health treatment may be useful in promoting adherence to interventions (i.e. effectiveness research). Dropout rates are traditionally high for PA-based interventions in populations with mental health issues [98•, 99•]⁠. RCTs that focus on determining the most engaging and effective PA protocol may help to increase adherence to an extent. For example, determining the minimum frequency or intensity of PA required to effectively reduce symptoms may be more acceptable amongst people who may exhibit symptoms that impair motivation [100]⁠.

Moreover, research is required to delineate the most significant factors that mediate adherence to PA interventions in people with anxiety disorders, such as intrinsic motivation. Research in people with serious mental illness has emphasised the importance of enhancing self-determined motivation through providing autonomy, competence and relatedness through PA [101•]⁠. Previous research in people with depression and schizophrenia have also identified the inclusion of a credible, appropriately qualified fitness professional leading the intervention, as another mediator of adherence [98•, 99•]⁠.

Future research should consider using theory-based mixed methods to investigate mediating factors that may be specific to individuals with anxiety disorders and can inform the development of interventions that are more engaging and acceptable to participants. It would also be beneficial for this research to focus more broadly on developing constructive frameworks through which PA-based interventions can be prescribed in mental health settings. This would help to reduce the practical barriers to implementation and increase the availability of PA-based therapy to people with anxiety disorders.

While this paper has predominantly been indiscriminate in discussing anxiety disorders, there may be differences between how people with different types of anxiety disorders engage with PA. For example, people with social anxiety disorders may be more reluctant to attend PA interventions that are delivered in group settings than those whose anxiety is not derived from social situations to the same extent. There is a lack of RCTs comparing the response of people with different types of anxiety disorders to PA [32••]⁠ or in the impact of related psychiatric problems (e.g. substance misuse).

Research into the effectiveness of PA at reducing anxiety symptoms has accumulated a strong evidence base, but more work is needed to identify methods of understanding and promoting adherence to interventions. This also includes the development of novel platforms to increase enjoyment of PA as a mechanism for maintaining higher adherence. A recent example of this is *exergaming,* which refers to the integration of action video games with PA and is being developed for people with schizophrenia [102]⁠.

## Physical Fitness and Anxiety Disorders

The relationship between physical fitness and anxiety is also unclear. Few studies have sufficiently accounted for baseline physical fitness or changes in physical fitness in their analysis [32••, 46••]⁠. This is problematic as it has led to an uncertainty over the necessity for physical fitness to change for a PA-based intervention to be effective. Some evidence suggests that PA-based interventions have been effective in reducing anxiety symptoms without any changes in physical fitness [32••]⁠. It is also unclear whether baseline physical fitness is a mediating factor between PA and anxiety symptoms. Two studies have found that participants with lower levels of cardiorespiratory fitness show the greatest improvements in symptoms of anxiety [92•, 103•]⁠. Differentiating the anxiolytic effects of PA independent of the role of physical fitness will be essential to inform how PA should be administered amongst different groups and how to tailor the focus of such efforts. For example, it is possible that standalone PA interventions with a focus on fitness-enhancing exercise may be best suited for populations with low base levels of physical fitness, but as an adjunction for people with higher levels of physical fitness.

Measuring changes in physical fitness will also be important in determining whether PA-based interventions can reduce the physical health risks associated with anxiety disorders. There is a notable lack of research that directly assesses the impact of PA on the physical health of people with anxiety disorders [46••]⁠. The elevated risk of cardiovascular disease, high blood pressure and premature mortality in people with anxiety disorders [9, 10•, 12, 13]⁠ is representative of a wider problem in mental health. Mental illness is broadly associated with poorer physical health outcomes; the severity of which is epitomised by a 15- to 20-year mortality gap between individuals with serious mental illness and the general population [104••, 105, 106]⁠. Factors such as physical inactivity represent a significant, but modifiable cardiovascular risk factor that is widespread in people with mental health problems [107]⁠, including anxiety disorders [25•, 53]⁠. Research in people with serious mental illness has shown PA-based interventions can improve physical health [28, 43]⁠ and may have a similar effect in people with anxiety disorders.

Including objective measures of physical fitness such as VO_2_ max tests, as well as indicators of physical health such as cardiovascular or pulmonary assessments at pre- and post-intervention time points, would help to determine whether PA can improve physical health in people with anxiety disorders. It would also be beneficial for high-quality RCTs to be conducted that directly assess the impact of PA-based interventions on the physical health of people with anxiety disorders as a primary outcome measure. Such research is important for the wellbeing of those with anxiety disorders, but would also help to address the stark health inequalities associated with mental illness more broadly, which appears to be worsening [104••]⁠.

## Conclusion

There is a growing basis of evidence to suggest that PA is associated with reduced symptoms of anxiety in the general population and can be used to treat symptoms of anxiety in those with anxiety disorders. Moreover, PA may have further benefits to physical health and wellbeing. However, despite the physical health burden in people with anxiety disorders, relatively, little is known about the benefits of PA on physical health and cardiovascular disease in this population. The underlying mechanisms for the effects of PA on anxiety symptoms have not been elucidated. Similarly, research assessing the optimal PA protocols for reducing symptoms or on promoting adherence to PA have been lacking. Addressing these gaps in the literature will be important in developing efficacious and implementable PA-based interventions.

It will also be necessary to establish where PA would be best integrated into the current service model. One option would be to take a step-wise approach by including lifestyle approaches such as PA as an early intervention to treat mild symptoms of anxiety, precluding the use of CBT or pharmacotherapy [108••]⁠. The wider benefits of PA to patient wellbeing, and particularly to physical health, may also justify the inclusion of PA as an adjunction to treatment for cases where symptoms are moderate or severe.

Furthermore, population-level data demonstrates that the relationship between PA and anxiety extends to non-clinical groups, suggesting that promoting PA could be used as a universal approach for the primary prevention of anxiety [25•]⁠. Promoting PA has already been suggested as a promising preventative strategy for other mental health conditions such as depression [109]⁠ and may represent a cost-effective and practical method of reducing the prevalence of anxiety and anxiety-related disorders. However, more research in non-clinical population groups would be needed to confirm this.

More RCTs must be conducted to establish the most effective PA intervention design and method of delivery for reducing anxiety symptoms and promoting adherence and physical health.
